# Müller Glia in Retinal Development: From Specification to Circuit Integration

**DOI:** 10.3389/fncir.2021.815923

**Published:** 2022-02-04

**Authors:** Joshua M. Tworig, Marla B. Feller

**Affiliations:** ^1^Department of Molecular and Cell Biology, University of California, Berkeley, Berkeley, CA, United States; ^2^Helen Wills Neuroscience Institute, University of California, Berkeley, Berkeley, CA, United States

**Keywords:** morphogenesis, neuronal-glial signaling, synaptogenesis, transcription factor, Notch

## Abstract

Müller glia of the retina share many features with astroglia located throughout the brain including maintenance of homeostasis, modulation of neurotransmitter spillover, and robust response to injury. Here we present the molecular factors and signaling events that govern Müller glial specification, patterning, and differentiation. Next, we discuss the various roles of Müller glia in retinal development, which include maintaining retinal organization and integrity as well as promoting neuronal survival, synaptogenesis, and phagocytosis of debris. Finally, we review the mechanisms by which Müller glia integrate into retinal circuits and actively participate in neuronal signaling during development.

## Introduction

Müller glia are the predominant synaptic astroglia of the retina. They tile the entire tangential retinal plane and exhibit a radial morphology which traverses from photoreceptors to inner limiting membrane. Müller glia exhibit a strikingly complex morphology, with distinct membrane specializations that contact photoreceptor outer segments, neuronal somata within nuclear layers, neurites within synaptic layers, and blood vessels throughout the retina. Their proximity to virtually every retinal cell type positions Müller glia ideally to guide the wiring and functioning of retinal circuits (Reichenbach and Bringmann, 2013).

Since the days of Heinrich Müller and Santiago Ramon y Cajal, who, respectively, discovered and later meticulously described the morphology of Müller glia and other astroglial cells (Müller, 1851; Ramon y Cajal, 1893), advances in genetic targeting as well as optical imaging have revealed essential roles for these cells in brain development and function. Müller glia, like other astroglial cell types, are born from the same neuroepithelial progenitors as those which produce neurons (Cepko et al., 1996). Müller glia are the only glial cell type to be born from progenitors within the retina. The two other retinal glial populations, astrocytes and microglia, migrate into the developing retina from other sources and play important developmental roles that are both distinct from and overlapping with those of Müller glia, as has been recently reviewed by other authors (Li et al., 2019; Dixon et al., 2021; Paisley and Kay, 2021).

Shortly following their birth, Müller glia begin to sculpt and integrate with nascent circuits as their morphological complexity grows and as they release molecules that are critical for neuronal survival and synaptogenesis (Reichenbach and Bringmann, 2013). As circuits mature in the retina and elsewhere in the brain, astroglia dynamically interact with neuronal processes and synapses via direct physical contact and signaling through an array of neurotransmitter receptors, ion channels, transporters, and exchangers expressed by glia (Lavialle et al., 2011; Rosa et al., 2015; Zhang et al., 2019). This enables Müller glia and other synaptic glial cells to integrate and respond to neuronal signaling across a range of time scales, often involving intracellular mobilization of second messengers such as calcium, as well as changes in gene expression and morphology (Uckermann et al., 2004; Rillich et al., 2009; Hasel et al., 2017).

Much effort has been focused on understanding the developmental trajectory and functions of astrocytes within brain circuits (Allen and Lyons, 2018), while less is known about the role of Müller glia in retinal circuit development. Here, we provide an overview of the current understanding of Müller glial development. We begin by describing the signaling events underlying Müller glial specification, patterning, and differentiation, and we move on to discuss their known roles in promoting retinal neuronal survival, synaptogenesis, and synapse pruning. Finally, we review the mechanisms by which Müller glia integrate into developing circuits, enabling them to rapidly respond to synaptic activity within the first few days after their exit from the cell cycle. We also note that although many recent studies have indicated a role for Müller glia in retinal regeneration by reprogramming gene expression (Goldman, 2014; Blackshaw and Sanes, 2021; Too and Simunovic, 2021) this will not be a focus of the present review.

## Development of Müller Glial Cells

### Fate Determination and Differentiation

**FIGURE 1:**
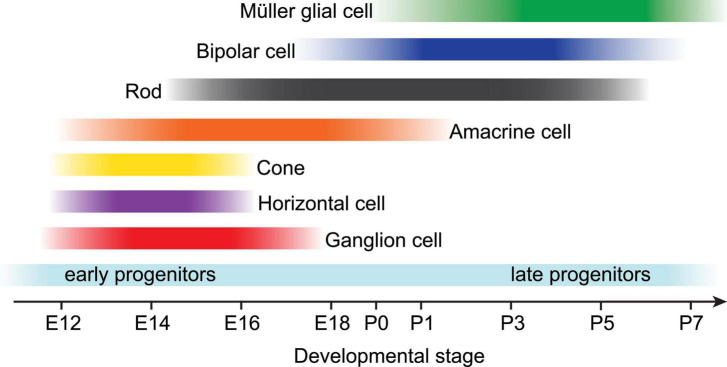
Developmental timeline of retinal neuroepithelium-derived cell birth in rodents. Near the end of embryonic development (starting around E12 in rodents), multipotent progenitor cells become competent to produce all retinal neurons and Müller glia in a conserved, overlapping temporal sequence. Early retinal progenitors first produce ganglion cells, horizontal cells, cone photoreceptors, and most amacrine cells prior to birth. Postnatally, late retinal progenitors produce most rod photoreceptors, bipolar cells, and lastly Müller glia. Adapted from Bassett and Wallace (2012).

Before Müller glial fate is specified among progenitor cells, the vertebrate retina has already completed much of its anatomical development; neuronal fate specification is complete, the cellular and plexiform layers have formed, and synapses are undergoing active refinement at this time. During vertebrate embryogenesis, the developing retina consists of a pool of mitotic, multipotent progenitor cells which arise from the neuroepithelium and eventually produce all classes of retinal neurons and Müller glial cells (Figure 1). These cell classes are specified in an orderly but overlapping sequence which is generally conserved across vertebrate species; ganglion cells are born first during late embryogenesis, followed by amacrine cells, horizontal cells, and cone photoreceptors. Rod photoreceptors differentiate over an extended period of time through the first postnatal days in mice, followed shortly thereafter by bipolar cells and Müller glia (Young, 1985; La Vail et al., 1991; Prada et al., 1991; Cepko et al., 1996; Wong and Rapaport, 2009).

**FIGURE 2:**
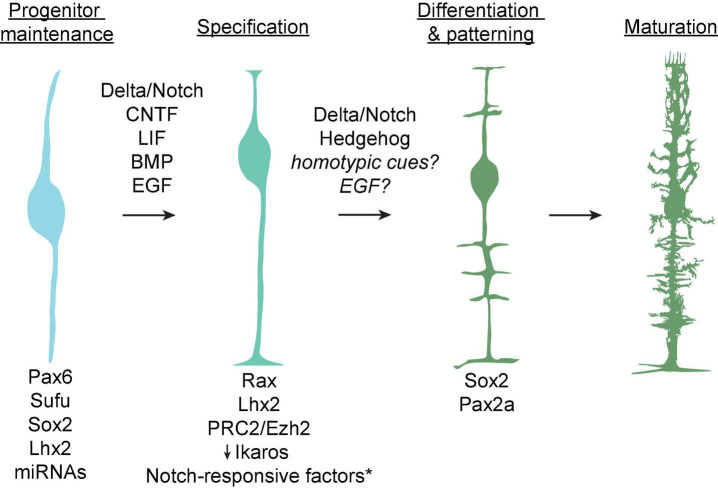
Known factors underlying the progression from retinal progenitors to mature Müller glia. Cell intrinsic molecules including transcription factors and microRNAs which mediate maintenance of cell identity or differentiation at each step are listed at bottom. Contact-dependent (Delta/Notch) and growth factor signaling pathways thought to promote Müller glial specification and differentiation are listed above arrows. *Notch-responsive factors include Hes1, Hes5, Hesr2, Id1-3, Sox9, and Nfia/b/x.

These cell fate determinations are coordinated by transcriptional regulatory networks which generate distinct competence states wherein progenitor cells may produce a subset of postmitotic retinal cells (Figure 2). A long history of study using lineage tracing and genetic perturbation has implicated a number of transcription factors required for the maintenance of various progenitor competence states, including Pax6 (required for maintenance of multipotency), Sufu (a negative regulator of hedgehog signaling-induced gene expression), and Sox2 (downstream of hedgehog signaling and required for neurogenesis) (Bassett and Wallace, 2012). Additionally, the conserved transcription factor Ikaros confers competence among progenitors to produce early-born neuronal types, and its downregulation is required for transition into a competence state in which Müller glia are produced (Elliott et al., 2008). Conversely, upregulation of the homeobox gene Rax induces competence among late progenitors to produce Müller glia (Furukawa et al., 2000).

### Extrinsic Factors Affecting Müller Glial Development

Within these competence states, cell extrinsic signaling regulates fate choice via contact-mediated signaling or release of soluble factors from neighboring cells (Livesey and Cepko, 2001; Bassett and Wallace, 2012; Cepko, 2014) (Figure 2). In particular, the Delta-Notch transmembrane signaling pathway is instructive for cell fate choice throughout development of the retina and the rest of the brain (Jadhav et al., 2006; Louvi and Artavanis-Tsakonas, 2006). Several studies across multiple vertebrate species have shown that activation of Notch in late retinal progenitors promotes expression of Müller glial markers, in some cases at the expense of differentiated neurons (Henrique et al., 1997; Zheng-Zheng and Cepko, 1997; Furukawa et al., 2000; Scheer et al., 2001). This occurs via interactions between the Notch pathway and a variety of gene regulatory networks which include the transcription factors Lhx2, Sox9, and the basic helix-loop-helix (bHLH) superfamily. First, Lhx2 directly activates genes in the Notch pathway to induce proliferation among gliocompetent retinal progenitors. Lhx2 remains highly expressed in glial precursors and is required for their terminal differentiation and expression of Müller glia-specific markers including p27^*Kip*1^, glutamine synthetase, and Sox9 (Melo et al., 2016a,b). Subsequently, Sox9 functions in late progenitors either to induce a gliocompetent state or to promote terminal differentiation into Müller glia (Kohn et al., 2015; Poché et al., 2008). Finally, a network of bHLH factors including Hes1, Hes5, and Hesr2 positively regulate Müller glial fate choice downstream of Notch activation by dimerizing and binding to regulatory elements of proglial genes (Furukawa et al., 2000; Hojo et al., 2000; Satow et al., 2001). These function in concert with non-DNA binding bHLH factors known as Inhibitor of differentiation 1–3 (Id1-3), which promote cell cycle progression among Müller glial progenitors by heterodimerizing with and sequestering proneuronal bHLH factors, thereby preventing their binding to DNA (Lasorella et al., 2002; Uribe and Gross, 2010; Mizeracka et al., 2013). Thus, the bHLH pathways interact with and modulate each other to coordinate Müller glial cell fate determination downstream of Notch activation.

In addition to contact-mediated signaling through Notch, several growth factor signaling pathways have been shown to promote Müller glial fate (Figure 2). These include ciliary neurotrophic factor (CNTF) and leukemia inhibitory factor (LIF), which promote Müller glial genesis by activating the Janus kinase (JAK)-signal transducer and activation of transcription (STAT) and extracellular signal-regulated kinase (ERK) pathways involved in cell growth and proliferation (Goureau et al., 2004; Todd et al., 2016). Transient expression of bone morphogenetic protein (BMP) near the end of retinal neurogenesis leads to phosphorylation of Smad1/5/8 in prospective Müller glial cells, leading to upregulation of Müller glia-specific genes (Ueki et al., 2015). Similarly, epidermal growth factor (EGF) promotes Müller glial fate using a mechanism that interacts with BMP signaling, and progenitor competence to produce Müller glia in response to EGF begins around P0 in rodents (Lillien, 1995; Lillien and Wancio, 1998; Close et al., 2006; Ueki and Reh, 2013). On the other hand, transforming growth factor β (TGFβ) secreted from neurons inhibits proliferation of Müller glial precursors in a manner that is antagonistic with EGF signaling (Close et al., 2005). This occurs via activation of the cyclin-dependent kinase inhibitor p27*^Kip1^*, which is required for cell cycle withdrawal in Müller glial precursors (Levine et al., 2000).

Despite the progress made toward defining the regulatory networks underpinning Müller glial fate determination, this is still a poorly understood process. Gain- and loss-of function studies have identified a variety of pro-glial and pro-neuronal bHLH transcriptional regulators under the control of Notch. However, the effects of Notch signaling are highly pleiotropic, resulting in a range of activities that either repress or promote progenitor differentiation depending on developmental timepoint, cell type, and transcriptional cross-talk with other signaling pathways such as JAK-STAT (Louvi and Artavanis-Tsakonas, 2006). Although this complicates our understanding of Müller glial differentiation, advances in single cell RNA profiling enable pseudo-tracking of transcriptomes among all neural cell types across development. This methodology is currently being used to discover novel regulators of cell fate using differential expression analysis (Shekhar and Sanes, 2021). For example, a recent study using single-cell RNA-sequencing revealed that the Notch-regulated nuclear factor 1 (NF1) transcription factors Nfia/b/x are enriched in late retinal progenitors and promote the formation of Müller glia and bipolar cells (Clark et al., 2019).

### Roles of Epigenetics and miRNAs in Müller Glial Development

Transcriptional regulatory networks function in an environment in which chromatin varies in its accessibility depending on covalent modifications to histones or DNA. Recent studies using epigenomic tools such as ChIP-Seq have pointed toward histone modification as a mechanism of Müller glial specification and differentiation. Polychrome repressive complex 2 (PRC2) acts as a repressor of gene expression by transferring methyl groups to lysine 27 on histone 3 (H3K27me2/3) (Hansen et al., 2008). Inactivation of the histone methyltransferase Ezh2, a component of PRC2, leads to enhanced and premature specification of Müller glia at the expense of late born neuronal types, possibly caused by downregulation of proneural factors. Ezh2 knockout retinas display enhanced expression of markers for mature Müller glia, along with glial membrane disorganization and upregulation of GFAP, suggesting that Ezh2 also functions as a regulator of differentiation in these cells (Iida et al., 2015; Zhang et al., 2015). RNA-sequencing reveals that the effects of Ezh2 are due in part to dysregulation of the Müller glia-promoting gene *Hes1*, which is normally targeted for H3K27me3 modification by PRC2/Ezh2, thereby dampening glial differentiation under normal conditions (Ueno et al., 2017).

A series of studies has also implicated microRNAs (miRNAs) in regulating the timing of Müller glial specification or differentiation via translational silencing of various progenitor genes. Initial evidence in support of this hypothesis came from conditional knockout studies which abolished the function of the RNA processing enzyme Dicer, halting the maturation of miRNAs during retinal development. This resulted in agenesis of Müller glia associated with perturbed expression of Notch-responsive genes and loss of the pro-glial transcription factor Sox9, as well as defects in specification of some neuronal types (Georgi and Reh, 2010; Davis et al., 2011). RNA expression studies have identified specific miRNAs that are upregulated or downregulated during retinal progenitor differentiation. Microarray expression data across development along with targeted knock-down of miRNAs were used to identify three targets of Dicer – let-7, miR-125, and miR-9 – as key factors in the specification of late progenitors which go on to produce Müller glia (La Torre et al., 2013). Another example is the highly conserved miR-7a, which directly binds Notch3 mRNA and suppresses its expression. This prevents differentiation of retinal progenitors into Müller glia, as evidenced by perturbed expression of the glial markers glutamine synthetase and cyclin D3 following miR-7a misexpression. This occurs without interfering with specification or proliferation of other cell types, suggesting that in the retina, the miR-7a/Notch3 system functions primarily in Müller glia differentiation (Baba et al., 2015). For a comprehensive review of the known miRNAs affecting Müller glial specification or differentiation, see Quintero and Lamas (2018).

Further study aimed at unraveling the interactions between Müller glial epigenome and transcriptome during differentiation, along with expanded access to new sequencing methods and the data they produce will provide necessary detail in our understanding of Müller glial specification and differentiation.

### Müller Glial Patterning and Morphogenesis

**FIGURE 3:**
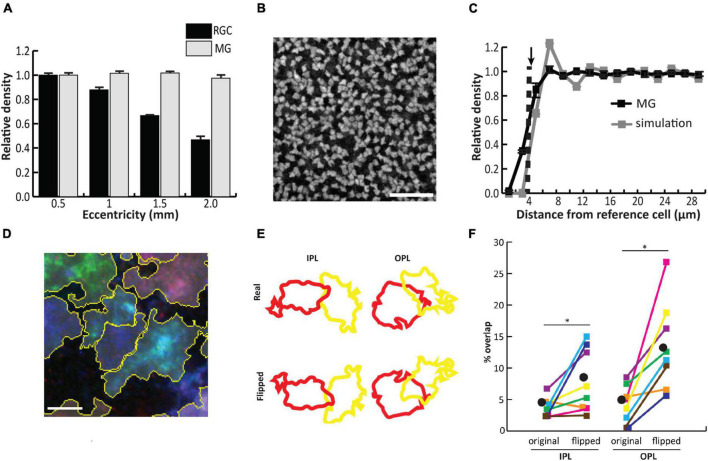
Müller glial (MG) cell bodies are uniformly distributed across the mouse retina and randomly arranged in the tangential plane, while processes in synaptic layers tile the retina with minimal overlap. **(A)** In contrast to retinal ganglion cells, MG are uniformly distributed across all retinal eccentricities. **(B)** Staining for the glial marker Sox9 in the inner nuclear layer (INL) reveals tight packing of cell bodies separated by small gaps. **(C)** Density recovery profile (DRP) for MG cell bodies (black) reveals an exclusion zone which matches the average cell body size. DRP for simulated random arrays (gray) matched in cell size and density to MG corresponds well with the true MG DRP, suggesting random assortment of cell bodies in the INL. **(D)** Combinatorial expression of multiple fluorescent markers via the Brainbow system followed by contiguous spatial segmentation delineates the territories of individual cells within the inner and outer plexiform layers (IPL/OPL). **(E)** Arbor overlap between neighboring pairs of MG was calculated before and after flipping each image about its horizontal axis. **(F)** Arbor overlap is significantly greater when territories are flipped, suggesting non-random arrangement. Scale bars (in μm): 50 **(B)**, 5 **(D)**. Adapted from Wang et al. (2017).

By the time of Müller glial differentiation, around postnatal day 5 (P5) in mice, anatomical development of the retina is nearly complete, with most neuronal types already present across three nuclear layers and two nascent synaptic layers (Hoon et al., 2014). Components of the Notch signaling pathway remain highly expressed in Müller glia for an extended period following cell cycle exit, and are required for maintenance of Müller glial identity during this time (Nelson et al., 2011). Initially, Müller glia closely resemble retinal progenitor cells, with simple apical and basal processes projecting from the cell body, which is located near the middle of the inner nuclear layer (INL) following interkinetic nuclear migration from the apical region (MacDonald et al., 2015) (Figure 2). Müller glia are uniformly distributed across the retina (Figure 3A), and the spatial organization of their cell bodies within the INL is random, seemingly only constrained by physical restrictions from nearby cells (Figures 3B,C) (Wang et al., 2017). This stands in contrast to some retinal neurons like horizontal cells and starburst amacrine cells, which form mosaics of regularly spaced cell bodies (Reese and Keeley, 2015; Keeley et al., 2020).

Despite the random arrangement of Müller glial cell bodies within the INL, their apical and basal processes, which project through the outer and inner plexiform layers (OPL/IPL), outer nuclear layer (ONL), and outer/inner limiting membranes (OLM/ILM), are arranged in a semi-regular array within the tangential retinal plane (Franze et al., 2007). Müller glia are generally regarded as a homogeneous cell type in mice and primates based on gene expression analysis (Shekhar et al., 2016; Wang et al., 2017; Peng et al., 2019; Yan et al., 2020). In developing chicken retina, however, Müller glia comprise multiple clusters defined by retinal position-dependent expression of various markers, some of which follow a progressive developmental restriction to either central or peripheral retina (Yamagata et al., 2021). The regular spacing of Müller glial stalks seems to be governed by homotypic interactions between their processes which begin extending laterally into synaptic layers around P6 in the mouse. Using the ‘Brainbow’ approach to stochastically label neighboring Müller glia with different colored fluorescent reporters, it was found that Müller glial lateral processes tile across the OPL and IPL, with minimal overlap between arbors of neighboring cells (Wang et al., 2017) (Figures 3D–F). The hypothesis that homotypic repulsive interactions between Müller glial processes enable regular tiling is supported by laser ablation experiments in which individual Müller glial cells were removed from the embryonic zebrafish retina, resulting in a region of OPL devoid of glial processes. This region was subsequently filled in by neighboring intact Müller glial processes, suggesting a temporary relief of glia-glia interactions that normally prevent process overlap between neighbors (Williams et al., 2010).

Molecules mediating homotypic interactions between Müller glia have not yet been identified. Among astrocytes of the *Drosophila* ventral nerve cord, tiling, infiltration of neuropil, and domain size are controlled by fibroblast growth factor (FGF) secretion from neurons and subsequent signaling through the astrocytic FGF receptor Heartless (Htl) (Stork et al., 2014). The cell adhesion molecule Lapsyn acts downstream of or in parallel with FGF signaling to control astrocytic branch morphogenesis (Richier et al., 2017). In mouse cortical astrocytes, the cell adhesion molecule hepaCAM plays a similar role in branch outgrowth, in part by stabilizing connexin 43 (Cx43) to promote astrocytic coupling (Baldwin et al., 2021). Whether these factors influence Müller glial tiling is not known but represent interesting avenues of future research.

**FIGURE 4:**
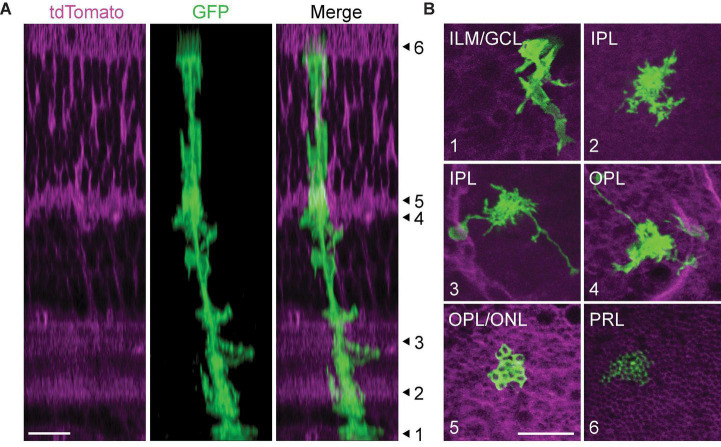
Morphological compartments of mature Müller glia. **(A)** Orthogonal projection of a 2-photon volumetric image from mature GLAST/mTmG mouse retina. This mouse line expresses membrane-bound tdTomato ubiquitously, and sparse activation of Cre recombinase induces expression of membrane-bound GFP in a subset of Müller glia. Numbered arrowheads correspond to focal planes shown in **(B)** to highlight different morphological compartments. **(B)** (1) Müller glial endfeet in the inner limiting membrane (ILM) and ganglion cell layer (GCL). (2) Lateral processes in the inner plexiform laer (IPL). (3) Lateral processes and long-ranging blood vessel-contacting processes in the IPL. (4) Lateral processes and blood vessel-contacting processes in the outer plexiform layer (OPL). (5) Photoreceptor terminal-ensheathing processes at the OPL-outer nuclear layer border. (6) Müller glial microvilli extending between photoreceptor inner segments. PRL: photoreceptor layer. Scale bars 10 μm. From Tworig, unpublished images.

As Müller glia mature, they undergo dramatic morphological change across the retina: basal processes form branching endfeet which ensheathe neuronal somata in the ganglion cell layer (GCL); fine lateral processes ramify throughout the synaptic layers; apical processes wrap around photoreceptor cell bodies in the ONL and project finger-like microvilli through the OLM to contact photoreceptor outer segments; and processes extend throughout the retina to specifically contact blood vessels and mediate neurovascular coupling (Figures 4A,B). Within the IPL, lateral processes exhibit sublaminar-specific outgrowth, with denser ramification in sublayers S1, S3, and S5 than in the choline acetyltransferase-positive (ChAT+) sublayers S2 and S4 (Reichenbach and Reichelt, 1986; Metea and Newman, 2006; Wang et al., 2017; Tworig et al., 2021).

The signaling mechanisms underlying outgrowth and spacing of Müller glial processes remain unknown, although hedgehog signaling originating from retinal ganglion cells may play a role in setting up their orientation (Wang et al., 2002). In addition to its previously mentioned role in glial specification, the hedgehog-responsive transcription factor Sox2 is required in Müller glia for maturation of their basal endfeet and lateral processes in synaptic layers (Bachleda et al., 2016). A possible mechanism for Sox2-mediated morphogenesis which has yet to be explored implicates EGF receptor (EGFR) activity, which is directly upregulated by Sox2 (Hu et al., 2010) and overlaps with the period of Müller glial process outgrowth in rodents (Close et al., 2006; Wang et al., 2017). Chronic manipulations of EGF signaling have revealed its role in glial fate determination as previously described (Lillien, 1995; Lillien and Wancio, 1998; Ueki and Reh, 2013; Sardar Pasha et al., 2017), but EGFR activation also induces cytoskeletal rearrangements and membrane protrusions independent of transcriptional modulation (Xie et al., 1998). Exogenous EGF promotes migratory behavior of cultured Müller glial cells (Pena et al., 2018) and slightly enhances motility of Müller glial processes during development (Tworig et al., 2021), but further study is needed to determine whether endogenous EGFR signaling is instructive for glial process outgrowth during normal retinal development.

Due to the stereotyped nature of Müller glial morphogenesis across retinal layers, as well as the genetic and experimental tractability of the retina, the cellular and molecular events underlying process outgrowth are investigable using genetic screening and imaging approaches. This was recently done using a CRISPR-based reverse genetic screen, which identified 41 genes implicated in various aspects of Müller glia morphogenesis in zebrafish (Charlton-Perkins et al., 2019). These genes affected distinct morphological features of Müller glia, including cell body position (examples: Nav1b, Lamb4, and Timp2b), tiling across the tangential plane (Nav1b, F8, Icn2, and Cdhr1), and lateral outgrowth of processes within the IPL and OPL (Nav1b, Mapa1b, Fat1b, Egr1, Slitrk2, and Dcaf8). In general, genes that are upregulated early during Müller glial differentiation affect broad aspects of morphogenesis, while genes upregulated later have more restricted effects on individual aspects of morphology. Many of these genes are conserved among glia in other species. One example is Pax2a, which is expressed by many glial cell types and is known to modulate cell morphology, adhesion, and differentiation (Charlton-Perkins et al., 2011). In zebrafish retina, Pax2a-mutant Müller glia display aberrant morphological characteristics in every retinal layer, including defects in tiling and outgrowth of processes into synaptic layers (Charlton-Perkins et al., 2019).

These studies of Müller glial morphogenesis in mouse and zebrafish were carried out by examining glial morphology in fixed samples. A better understanding of the factors involved in Müller glial process outgrowth and distribution will be achieved by carrying out live imaging of labeled Müller glia during retinal development. To this end, several studies have revealed that vertebrate Müller glia exhibit dynamic branching as they mature, with processes in synaptic layers undergoing extensions, retractions, sprouting, and elimination on the timescale of minutes as they sample space across the OPL and IPL (Williams et al., 2010; MacDonald et al., 2015; Tworig et al., 2021). This motility halts shortly after the onset of vision, suggesting that it is developmentally regulated or suppressed by synaptic activity. The potential roles for synaptic activity in Müller glial development are discussed in greater detail below.

## Roles of Müller Glia in Retinal Development

Throughout the developing central nervous system, astroglia broadly influence key events including neuronal growth and maturation, synapse formation and pruning, and critical period timing (Allen and Lyons, 2018; Ackerman et al., 2021; Ribot et al., 2021). Similarly, Müller glia affect many aspects of retinal development ranging from its overall cytological organization to the formation of specific visual circuits.

### Maintenance of Retinal Organization

**FIGURE 5:**
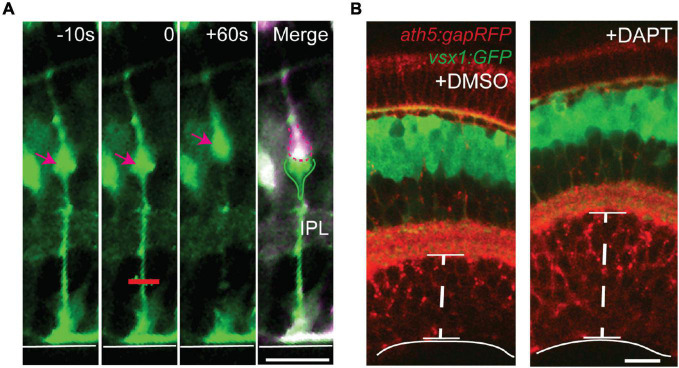
Müller glia act as springs to maintain strength and integrity of the retina. **(A)** Laser ablation was used to sever the basal processes of GFAP:GFP Müller glia from zebrafish *in vivo*. After ablation at *t* = 0 (red line), the basal process retracts and the cell body (arrow) is displaced apically, suggesting that Müller glia are under tension during development. **(B)** Retinal development in the absence of Müller glia following treatment with the Notch pathway inhibitor DAPT leads to expansion of the ganglion cell layer (GCL) without an increase in cell number, suggesting that tension along Müller glia promotes integrity of the GCL. Retinal neurons are labeled with membrane RFP using *ath5:gapRFP*, and bipolar cells are labeled with GFP using *vsx1:GFP*. Scale bars 15 μm. Adapted from MacDonald et al. (2015).

Müller glia are essential for the guidance and maintenance of cellular organization across the retina. This is evidenced by knockout studies in which retinas with perturbed Müller glial development exhibit lamination defects and form ectopic neural rosettes (Wang et al., 2002; Bachleda et al., 2016; Wohl et al., 2017). Disruption of Müller glial function with the glia-specific toxin α-aminoadipic acid prior to photoreceptor maturation abolishes contacts between Müller glial microvilli and photoreceptors and leads to aberrant migration of photoreceptors toward the subretinal space (Rich et al., 1995). Absence of Müller glia during zebrafish development results in retinal disorganization characterized by a propensity for tearing and aberrant photoreceptor spacing, possibly due to a reduction in tensile strength normally provided by Müller glia during retinal maturation via expression of various ECM-interacting proteins such as integrins (Guidry et al., 2003; MacDonald et al., 2015; Nagashima et al., 2017) (Figures 5A,B). Strikingly, however, Randlett et al. (2013) found that gross laminar organization and synaptic structures are maintained in zebrafish retinas devoid of Müller glia following pharmacological interference of Notch signaling, suggesting that neurons are able to form a rudimentary neuropil and layered structure autonomously.

### Secretion of Neurotrophins

As the retina matures, Müller glia secrete a variety of trophic factors that affect neuronal survival, promote neuritogenesis, or protect against excitotoxicity. This was initially determined by observing enhanced neuronal growth and neurite elongation among retinal neurons when cultured with Müller glia or Müller glia-conditioned media (Raju and Bennett, 1986; García et al., 2002). Subsequent study implementing Müller glia-neuron co-culture has identified a panel of Müller glia-derived growth factors or cytokines that mediate survival (examples: pigment-epithelium derived factor/PEDF, vascular endothelial growth factor/VEGF) (Saint-Geniez et al., 2008; Bai et al., 2009; Unterlauft et al., 2014) or neurite outgrowth (CNTF, interleukin-6/IL-6) (Yoshida et al., 2001) among retinal neurons. Co-culture experiments have also implicated glial ATP release as a promoter of neuritogenesis among RGCs via activation of P2Y_6_ receptors (Taguchi et al., 2016). Some of these signaling pathways work in concert with Wnt/β-catenin signaling in Müller glia, which modulates secretion of known neurotrophins including CNTF and brain-derived neurotrophic factor (BDNF) (Patel et al., 2015; Musada et al., 2020).

Under certain experimental conditions, neuronal signaling has been shown to modulate Müller glial secretion of trophic factors. For instance, extracellular ATP can cause release of FGF from Müller glia downstream of glial P2Y receptors (Reichenbach and Bringmann, 2016), while glutamate enhances glial synthesis of a panel of neurotrophins including BDNF, glial-cell line derived neurotrophic factor (GDNF), and nerve growth factor (NGF) (Taylor et al., 2003). This is consistent with known roles of glial cells in preventing glutamate excitotoxicity. Furthermore, hypoxic regulation of VEGF release from Müller glia and astrocytes following neuronal activity is thought to promote retinal vascularization during development (Stone et al., 1995; Lange et al., 2012). Through another pathway independent of VEGF, Müller glia secrete the pro-angiogenic compound Norrin, which binds to Frizzled-4 receptors and Lrp5 co-receptors on endothelial cells within microvasculature to activate Wnt/β-catenin signaling, thereby promoting growth of intraretinal capillaries (Xu et al., 2004; Ye et al., 2009). Norrin also exhibits neuroprotective effects in the retina following excitotoxic damage and promotes dendritic development in cortical neurons following secretion from astrocytes (Seitz et al., 2010; Miller et al., 2019). Although Norrin is constitutively expressed in Müller glia, whether its secretion is modulated by neuronal activity is unknown. Interestingly, release of neurotrophins from Müller glia can be modulated by trophic factors released from activated microglia, but whether this is also a feature during development remains to be determined (Harada et al., 2002; Wang et al., 2011).

In recent years, studies aimed at identifying glia-derived trophic factors important during retinal development have shifted toward high-throughput approaches to screen the glial secretome for candidate molecules using liquid chromatography and/or mass-spectrometry, with subsequent testing of candidates for neurotrophic activity in cell culture or retinal explant. These studies have identified molecules including osteopontin, LIF, transferrin, basigin, clusterin, and C-X-C motif chemokine 10 (CXCL10) as neurotrophic factors that are secreted by Müller glia and exhibit neuroprotective effects on retinal neurons (Toerne et al., 2014; Ruzafa et al., 2018). Interpretation of these results is limited, however, due to loss of three-dimensional organization and known alterations in gene expression profiles of Müller glia upon dissociation and growth under culture conditions (Hauck et al., 2003; Wohl et al., 2016). Future experiments using *ex vivo* or intact retina in combination with targeted genetic manipulation of pathways of interest may confirm a role for these glia-derived candidate molecules in regulating retinal development.

### Promotion of Synapse Turnover

Müller glia maturation occurs during a period of robust synaptogenesis, positioning them to influence the formation or stability of new synapses via secretion of various molecules. Thrombospondins (TSPs) are synaptogenic factors that are secreted by astroglia and interact with gabapentin receptors (α2δ-1) on neurons (Eroglu et al., 2009). Müller glia express TSP1 and TSP2 with enrichment in the OPL and in specific sublayers of the IPL, and impaired secretion of TSPs during development disrupts synapse formation in both plexiform layers (Koh et al., 2018). Recent evidence indicates that TSP1 acts specifically on direction-selective ganglion cells (DSGCs) to promote excitatory synapse formation with their presynaptic partners, starburst amacrine cells (SACs), requiring both α2δ-1 receptor and β1-integrin expression in DSGCs for this activity. The same study revealed that TSP1 knockout also reduces inhibitory synapse formation throughout the IPL, although perhaps compensatory and subsequent to loss of excitation (Koh et al., 2019). This is reminiscent of the mouse striatum, where signaling through astrocytic GABA_*B*_ receptors during synaptic activity leads to activation of the G_*i*_ pathway and subsequent TSP1 secretion from astrocytes to promote synaptogenesis between corticostriatal axon terminals and medium spiny neuron dendritic spines (Nagai et al., 2019). Neuronal activity-evoked expression of glial TSPs and other synaptogenic cues possibly depends on glial calcium mobilization downstream of receptor activation (Tran and Neary, 2006; Farhy-Tselnicker et al., 2021). It will be interesting to find out whether a similar mechanism is at play in Müller glia, which undergo robust activity-evoked responses during retinal development as described further below (Rosa et al., 2015; Zhang et al., 2019).

There are likely other synaptogenic factors secreted by Müller glia that have yet to be characterized, given the large and growing list of astrocyte-secreted factors elsewhere in the brain (Tan et al., 2021). A number of these are expressed by Müller glia throughout development but have not been tested for a role in synaptogenesis. D-serine is a co-agonist of NMDA receptors, exhibits synaptogenic properties when released from astrocytes, and has been detected in developing and mature Müller glia along with serine racemase, the enzyme that produces D-serine (Schell et al., 1997; Williams et al., 2006; Diaz et al., 2007; Dun et al., 2008; Diniz et al., 2012). D-serine has also been reported to enhance expression of immediate early genes in Müller glia downstream of NMDA receptor activation and phosphorylation of cAMP response element binding protein (CREB) transcription factors, which are responsive to calcium and cAMP signaling (Lamas et al., 2007; Hasel et al., 2017). Modulation of glial gene expression following D-serine-enhanced NMDAR activation occurs in part via cytosolic translocation of the calcium-binding transcriptional modulator DREAM (downstream regulatory element antagonist modulator) and a reduction in its DNA-binding ability (Chavira-Suárez et al., 2008). Further study is necessary to determine whether Müller glial D-serine release also acts as a direct synaptogenic cue during retinal development *in vivo*.

It is possible that Müller glia also secrete anti-synaptogenic compounds to facilitate synapse turnover. SPARC/osteonectin secreted from astrocytes inhibits excitatory synapse formation in neuron-glia co-culture and in superior colliculus between RGCs and their targets (Kucukdereli et al., 2011; Albrecht et al., 2012). SPARC is also expressed by Müller glia throughout their development and into adulthood, suggesting a role in modulating synaptic development (Vincent et al., 2008). This story is perhaps complicated by the recent detection in the Müller glial secretome of the pro-synaptogenic SPARC-like protein 1 (SPARCL1/Hevin), whose synaptogenic activity is antagonized by SPARC. For a more complete list of neurotrophic and synaptogenic molecules potentially secreted by Müller glia, see Musada et al. (2020).

The influence of Müller glia on synapse formation or maturation likely varies by retinal circuit, neuronal type, and developmental context. In the developing chick retina, as-yet unidentified factors secreted by glia promote expression of M_2_ muscarinic acetylcholine receptors (mAChRs) on neurons, with minimal effect on expression of other mAChRs (Belmonte et al., 2000). In developing zebrafish OPL, although Müller glial lateral process elaboration and ensheathment of photoreceptor terminals overlap with the period of synaptogenesis, photoreceptor synapses develop normally and remain stable following laser ablation of Müller glial apical processes, suggesting that glial contact is dispensable for synaptogenesis in the outer retina (Williams et al., 2010). However, photoreceptor synapses were assessed in the context of localized ablation of a few Müller glial cells, and although this rules out a glial contact-dependent mechanism, a paracrine function for Müller glia in promoting synaptogenesis within the OPL is plausible. Consistent with this idea, Müller glial expression of the molecular scaffold harmonin during development is necessary for photoreceptor synaptic organization and function in zebrafish (Phillips et al., 2011). It is not yet known whether contact-dependent signaling from Müller glia contributes to synapse formation in the IPL.

Müller glia may also regulate neuronal development via phagocytosis of cellular debris including apoptotic cells and synapses. This has been shown using electron microscopy in developing chick retina which revealed electron-dense debris derived from degenerating cells (Hughes and Lavelle, 1975), as well as TUNEL staining in developing turtle and rat retina which revealed pyknotic bodies and cytoplasmic DNA within vimentin-positive glial processes (Egensperger et al., 1996; Francisco-Morcillo et al., 2004). This seems to occur in parallel with phagocytosis of cellular debris in developing retina by microglia (Marín-Teva et al., 1999). The mechanisms underlying recognition and phagocytosis of cellular debris by Müller glia during development are unknown, but in mature retina phagocytosis of photoreceptor debris after injury requires the phagocytic pathway components Rac1 GTPase and phosphatidylserine (Nomura-Komoike et al., 2020). Phagocytic activity among Müller glia during development may be mediated by the receptor tyrosine kinase Mertk, the loss of which leads to cell-autonomous morphological alterations in Müller glia and reduced synapse density in postnatal retina, as well as reduced synaptogenic potential among cultured Müller glia (Scott et al., 2001; Koh et al., 2018).

**FIGURE 6:**
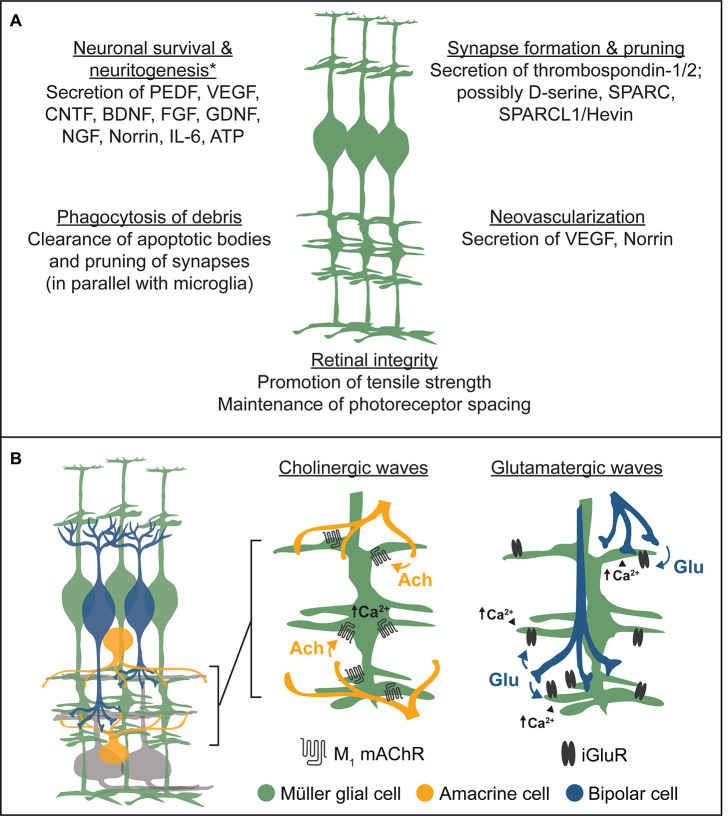
Summary of the roles of Müller glia in retinal development. **(A)** Müller glia engage in broad aspects of retinal development through the release of neurotrophins, synaptogenic proteins, and other trophic factors, while also clearing cellular debris and maintaining retinal integrity. Examples listed are non-exhaustive. *Müller glia-derived trophic factors promoting neuronal survival or neuritogenesis were primarily identified in neuron-glia coculture experiments. **(B)** Summary of Müller glial responses to cholinergic and glutamatergic retinal waves. *Left*, schematic diagram of basic inner retinal circuits showing bipolar cells (blue), Müller glia (green), amacrine cells (orange), and ganglion cells (gray) intermingling within the inner plexiform layer (IPL). *Middle*, during cholinergic retinal waves, starburst amacrine cells spontaneously release acetylcholine (ACh), which activates Müller glial M_1_ muscarinic acetylcholine receptors (mAChRs) following spillover from synapses. This induces calcium transients in Müller glia primarily localized to their stalks within the inner plexiform layer. *Right*, during glutamatergic retinal waves, bipolar cells spontaneously release glutamate (Glu), which activates Müller glial ionotropic glutamate receptors (iGluRs) following synaptic spillover, inducing calcium transients primarily localized to glial lateral processes within the IPL.

For a summary of the impacts of Müller glia on retinal development described above, see Figure 6A.

## Integration Into Circuits

### Participation in Patterned Activity

Müller glia are born and mature in an environment in which neural circuits are spontaneously active despite the absence of visual input. In many vertebrates, this activity is characterized by propagating waves of depolarization, termed retinal waves, which are critical for key aspects of visual system development such as eye-specific segregation and retinotopic refinement of retinogeniculate synapses (Blankenship and Feller, 2010; Arroyo and Feller, 2016; Kerschensteiner, 2016; Choi et al., 2021). Patterned spontaneous activity in the retina undergoes three distinct phases in mammals: stage 1 waves occur embryonically and are mediated by gap junctions and cholinergic signaling (Bansal et al., 2000), stage 2 waves are mediated by volume ACh release from SACs (Ford et al., 2012; Xu et al., 2016), and stage 3 waves are mediated by glutamate spillover from bipolar cells (Blankenship et al., 2009). In the days following their birth in mouse retina, Müller glia begin responding to cholinergic waves by undergoing calcium transients localized to their stalks within the IPL and mediated by glial M_1_ mAChRs (Rosa et al., 2015; Tworig et al., 2021). As waves shift from cholinergic to glutamatergic circuits, Müller glial lateral processes and stalks continue to undergo wave-evoked calcium transients via activation of ionotropic glutamate receptors (iGluRs) and M_1_ mAChRs (Figure 6B). This is associated with electrogenic glutamate uptake during each glutamatergic wave which slightly depolarizes Müller glia (Akrouh and Kerschensteiner, 2013).

Glial participation in developing visual circuits is a feature conserved across several species. In zebrafish retina, wave-evoked Müller glial calcium transients are thought to occur via a combination of glia-specific glutamate transporters and calcium-permeable AMPA receptors (Zhang et al., 2019). In *Xenopus* optic tectum, where correlated neural activity during development is driven by vision rather than spontaneous activity (Demas et al., 2012), radial astrocytes undergo calcium transients mediated by reversal of sodium-calcium exchangers (NCX) following light-evoked glutamate release from neurons and uptake by glia (Benfey et al., 2021). Glial participation in patterned activity within developing visual areas is not exclusive to vertebrates; in the developing *Drosophila* optic lobe, columnar astrocytes undergo phasic changes in intracellular calcium levels which are negatively correlated with nearby bursts of spontaneous neuronal activity (Akin et al., 2019).

### Activity-Dependent Glial Responses

The downstream effects of activity-evoked calcium signaling in Müller glia and other visual system astroglia have not been defined. One might infer based on studies in other systems that these responses could regulate glial morphology (Tremblay et al., 2009; Molotkov et al., 2013; Bernardinelli et al., 2014), gene expression (Farhy-Tselnicker et al., 2021), or release of synaptic or vascular modulators to guide circuit development and maintain homeostasis. A recent study from our lab explored the potential for activity-evoked Müller glial calcium transients to affect lateral process morphology and found that both intracellular calcium transients and neuronal activity are dispensable for motility of processes in the IPL, and that lateral process distribution is normal when retinal waves are perturbed (Tworig et al., 2021). These findings suggest that other undetermined functions of activity-evoked calcium signaling in Müller glia are at play during retinal waves. This contrasts with studies of perisynaptic brain astrocytes and *Xenopus* radial glia, which exhibit branch motility in response to neuronal activity (Tremblay et al., 2009; Molotkov et al., 2013; Bernardinelli et al., 2014).

Studies across brain regions have shown that neuronal activity can upregulate glial neurotransmitter transport, thereby tuning the fidelity of synaptic transmission via spatial buffering and preventing excitotoxic injury. This has been shown using cultured Müller glia, in which glutamate stimulation leads to upregulated expression of the glutamate transporter GLAST concomitantly with downregulation of glial NMDA receptors (Taylor et al., 2003; López-Colomé et al., 2016). It has been reported that NMDAR activation in Müller glia co-cultured with RGCs promotes RGC survival via upregulation of glial glutamate transport, thereby preventing excitotoxicity (Furuya et al., 2012). In rodent retina, GLAST is upregulated by Müller glia within the first two postnatal weeks (Pow and Barnett, 1999), suggesting a possible link between retinal wave-evoked Müller glial calcium transients and transporter expression.

Reflective of the bidirectional nature of neuron-glia signaling, Müller glial glutamate transport has been shown to tune the duration and frequency of retinal waves in zebrafish, and their blockade abolishes retinal waves completely, likely due to elevated extracellular glutamate concentration (Zhang et al., 2019). Similarly in developing *Xenopus*, blocking NCX-driven glial calcium transients during light-evoked activity leads to enhancement of neuronal responses, possibly resulting from impaired insertion of glutamate transporters into the glial membrane (Ibáñez et al., 2019; Benfey et al., 2021). In mouse retina, glutamate uptake in part by Müller glia seems to promote sequential activation of ON and then OFF RGCs during retinal waves (Akrouh and Kerschensteiner, 2013). Blockade of glutamate transport removes this sequential activation, inducing ON and OFF RGCs to fire synchronously during waves. This change in the spatiotemporal pattern of RGC activation has implications for development of independent downstream ON and OFF pathways, as observed in the ferret visual system (Lee et al., 2002).

In addition to upregulated glutamate transport, Müller glia undergo developmental changes in other membrane proteins capable of modulating neurotransmission or maintaining homeostasis. In rabbit retina, postnatal upregulation of the inward rectifier potassium channel Kir4.1 and aquaporin-4 accompany developmental changes in glial membrane properties including gradual hyperpolarization, reduced membrane resistance, increased capacitance, enhanced potassium conductance, and resistance to swelling under osmotic stress (Pannicke et al., 2002; Bosco et al., 2005; Wurm et al., 2006). Expression and subcellular localization of Kir4.1 and aquaporin-4 in Müller glia are governed in part by the extracellular cell adhesion molecule laminin (Hirrlinger et al., 2011). It is enticing to consider whether retinal waves play a role in this developmental increase of Kir4.1 and aquaporin-4 expression.

Along with possible modulation of membrane protein expression in Müller glia, retinal wave-evoked glial calcium transients may induce release of synaptic modulators. One fascinating example is the vision-dependent release from Müller glia of Acyl-CoA-binding protein (ACBP), which desensitizes GABA_*A*_ receptors (Barmack et al., 2004). Long-term horizontal optokinetic stimulation in rabbits enhances ACBP expression and phosphorylation, which increases its affinity for GABA_*A*_ receptors. This occurs only when stimulated in the preferred posterior-to-anterior direction and is associated with reduced gain of the optokinetic reflex. This is thought to result from reduced inhibitory effect of GABA on horizontal motion-preferring ON-OFF DSGCs following ACBP secretion (Barmack et al., 2004). ACBP production and phosphorylation in Müller glia has been shown to occur directly following stimulation with KCl and activation of protein kinase C (PKC) (Qian et al., 2008). As such, M_1_ mAChR-mediated PKC activation during cholinergic retinal waves could promote secretion of ACBP and influence the spatiotemporal properties of waves or the maturation of direction selective circuits by dampening the effect of GABA on postsynaptic neurons.

Neuronal activity may play a role in the release from Müller glia of any of the trophic molecules and synaptic modulators mentioned in previous sections, including thrombospondins and D-serine. As previously described, glutamate enhances secretion of neurotrophic factors such as BDNF and NGF from cultured Müller glia (Taylor et al., 2003), but whether this occurs *in vivo* is unknown. Future work employing targeted pharmacological and genetic perturbations along with selective sensors for synaptic modulators will aid in determining whether this is the case.

## Author Contributions

JT wrote the manuscript. MF made minor edits. Both authors contributed to the article and approved the submitted version.

## Conflict of Interest

The authors declare that the research was conducted in the absence of any commercial or financial relationships that could be construed as a potential conflict of interest.

## Publisher’s Note

All claims expressed in this article are solely those of the authors and do not necessarily represent those of their affiliated organizations, or those of the publisher, the editors and the reviewers. Any product that may be evaluated in this article, or claim that may be made by its manufacturer, is not guaranteed or endorsed by the publisher.

## References

[B1] Ackerman S. D. Perez-Catalan N. A. Freeman M. R. Doe C. Q. (2021). Astrocytes close a motor circuit critical period. *Nature* 592 414–420. 33828296 10.1038/s41586-021-03441-2 PMC9901311

[B2] Akin O. Bajar B. T. Keles M. F. Frye M. A. Zipursky S. L. (2019). Cell-type-Specific Patterned Stimulus-Independent Neuronal Activity in the Drosophila Visual System during Synapse Formation. *Neuron* 101 894.e–904.e. 30711355 10.1016/j.neuron.2019.01.008 PMC6437771

[B3] Akrouh A. Kerschensteiner D. (2013). Intersecting Circuits Generate Precisely Patterned Retinal Waves. *Neuron* 79:322. 10.1016/j.neuron.2013.05.012 23830830 PMC3737599

[B4] Albrecht D. López-Murcia F. J. Pérez-González A. P. Lichtner G. Solsona C. Llobet A. S. P. A. R. C. (2012). prevents maturation of cholinergic presynaptic terminals. *Mol. Cell. Neurosci.* 49 364–374. 10.1016/j.mcn.2012.01.005 22306863

[B5] Allen N. J. Lyons D. A. (2018). Glia as architects of central nervous system formation and function. *Science* 362 181–185. 10.1126/science.aat0473 30309945 PMC6292669

[B6] Arroyo D. A. Feller M. B. (2016). Spatiotemporal Features of Retinal Waves Instruct the Wiring of the Visual Circuitry. *Front. Neural Circuits* 10:54. 10.3389/fncir.2016.00054 27507937 PMC4960261

[B7] Baba Y. Aihara Y. Watanabe S. (2015). MicroRNA-7a regulates Müller glia differentiation by attenuating Notch3 expression. *Exp. Eye Res.* 138 59–65. 10.1016/j.exer.2015.06.022 26122050

[B8] Bachleda A. R. Pevny L. H. Weiss E. R. (2016). Sox2-Deficient Müller Glia Disrupt the Structural and Functional Maturation of the Mammalian Retina. *Invest. Ophthalmol. Vis. Sci.* 57 1488–1499. 10.1167/iovs.15-17994 27031842 PMC4819558

[B9] Bai Y. Ma J. Guo J. Wang J. Zhu M. Chen Y. (2009). Müller cell-derived VEGF is a significant contributor to retinal neovascularization. *J. Pathol.* 219 446–454. 10.1002/path.2611 19768732

[B10] Baldwin K. T. Tan C. X. Strader S. T. Jiang C. Savage J. T. Elorza-Vidal X. (2021). HepaCAM controls astrocyte self-organization and coupling. *Neuron* 109 2427.e–2442.e. 10.1016/j.neuron.2021.05.025 34171291 PMC8547372

[B11] Bansal A. Singer J. H. Hwang B. J. Xu W. Beaudet A. Feller M. B. (2000). Mice Lacking Specific Nicotinic Acetylcholine Receptor Subunits Exhibit Dramatically Altered Spontaneous Activity Patterns and Reveal a Limited Role for Retinal Waves in Forming ON and OFF Circuits in the Inner Retina. *J. Neurosci.* 20 7672–7681. 10.1523/JNEUROSCI.20-20-07672.2000 11027228 PMC6772851

[B12] Barmack N. H. Bilderback T. R. Liu H. Qian Z. Yakhnitsa V. (2004). Activity-Dependent Expression of Acyl-Coenzyme A-Binding Protein in Retinal Muller Glial Cells Evoked by Optokinetic Stimulation. *J Neurosci.* 24 1023–1033. 10.1523/JNEUROSCI.3936-03.2004 14762120 PMC6793587

[B13] Bassett E. A. Wallace V. A. (2012). Cell fate determination in the vertebrate retina. *Trends Neurosci.* 35 565–573. 22704732 10.1016/j.tins.2012.05.004

[B14] Belmonte K. E. McKinnon L. A. Nathanson N. M. (2000). Developmental expression of muscarinic acetylcholine receptors in chick retina: selective induction of M2 muscarinic receptor expression in ovo by a factor secreted by muller glial cells. *J. Neurosci.* 20 8417–8425. 10.1523/JNEUROSCI.20-22-08417.2000 11069949 PMC6773186

[B15] Benfey N. J. Li V. J. Schohl A. Ruthazer E. S. (2021). Sodium-calcium exchanger mediates sensory-evoked glial calcium transients in the developing retinotectal system. *Cell Rep.* 37:109791. 10.1016/j.celrep.2021.109791 34610307

[B16] Bernardinelli Y. Randall J. Janett E. Nikonenko I. König S. Jones E. V. (2014). Activity-dependent structural plasticity of perisynaptic astrocytic domains promotes excitatory synapse stability. *Curr. Biol.* 24 1679–1688. 10.1016/j.cub.2014.06.025 25042585

[B17] Blackshaw S. Sanes J. R. (2021). Turning lead into gold: Reprogramming retinal cells to cure blindness. *J. Clin. Invest.* 131:e146134. 10.1172/JCI146134 33529169 PMC7843217

[B18] Blankenship A. Feller M. (2010). Mechanisms underlying spontaneous patterned activity in developing neural circuits. *Nat. Rev. Neurosci*. 11, 18–29. 10.1038/nrn2759 19953103 PMC2902252

[B19] Blankenship A. G. Ford K. J. Johnson J. Seal R. P. Edwards R. H. Copenhagen D. R. (2009). Synaptic and extrasynaptic factors governing glutamatergic retinal waves. *Neuron* 62 230–241. 10.1016/j.neuron.2009.03.015 19409268 PMC2807181

[B20] Bosco A. Cusato K. Niccbia G. P. Frigeri A. Spray D. C. (2005). A Developmental Switch in the Expression of Aquaporin-4 and Kir4.1 from Horizontal to Müller Cells in Mouse Retina. *Invest. Ophthalmol. Vis. Sci.* 46 3869–3875. 10.1167/iovs.05-0385 16186376

[B21] Cepko C. (2014). Intrinsically different retinal progenitor cells produce specific types of progeny. *Nat. Rev. Neurosci.* 15 615–627. 10.1038/nrn3767 25096185

[B22] Cepko C. L. Austin C. P. Yang X. Alexiades M. Ezzeddine D. (1996). Cell fate deternination in the vertebrate retina. *Trends Neurosci.* 93 589–595. 10.1073/pnas.93.2.589 PMC40096 8570600

[B23] Charlton-Perkins M. Almeida A. D. MacDonald R. B. Harris W. A. (2019). Genetic control of cellular morphogenesis in Müller glia. *Glia* 67 1401–1411. 10.1002/glia.23615 30924555 PMC6563441

[B24] Charlton-Perkins M. Whitaker S. L. Fei Y. Xie B. Li-Kroeger D. Gebelein B. (2011). Prospero and Pax2 combinatorially control neural cell fate decisions by modulating Ras- and Notch-dependent signaling. *Neural Dev.* 6:20. 10.1186/1749-8104-6-20 21539742 PMC3123624

[B25] Chavira-Suárez E. Ramírez M. Lamas M. (2008). d-Serine/N-methyl-d-aspartate receptor signaling decreases DNA-binding activity of the transcriptional repressor DREAM in Müller glia from the retina. *Neurosci. Lett.* 432 121–126. 10.1016/j.neulet.2007.12.021 18191896

[B26] Choi B. Chen Y. Desplan C. (2021). Building a circuit through correlated spontaneous neuronal activity in the developing vertebrate and invertebrate visual systems. *Genes Dev*. 35, 677–691. 10.1101/gad.348241.121 33888564 PMC8091978

[B27] Clark B. S. Stein-O’Brien G. L. Shiau F. Cannon G. H. Davis-Marcisak E. Sherman T. (2019). Single cell RNA-Seq analysis of retinal development identifies NFI factors as regulating mitotic exit and late-born cell specification. *Neuron* 102:1111. 10.1016/j.neuron.2019.04.010 31128945 PMC6768831

[B28] Close J. L. Gumuscu B. Reh T. A. (2005). Retinal neurons regulate proliferation of postnatal progenitors and Müller glia in the rat retina via TGFβ signaling. *Development* 132 3015–3026. 10.1002/cne.22741 15944186

[B29] Close J. L. Liu J. Gumuscu B. Reh T. A. (2006). Epidermal growth factor receptor expression regulates proliferation in the postnatal rat retina. *Glia* 54 94–104. 10.1002/glia.20361 16710850

[B30] Davis N. Mor E. Ashery-Padan R. (2011). Roles for Dicer1 in the patterning and differentiation of the optic cup neuroepithelium. *Development* 138 127–138. 10.1242/dev.053637 21138975

[B31] Demas J. A. Payne H. Cline H. T. (2012). Vision Drives Correlated Activity without Patterned Spontaneous Activity in Developing Xenopus Retina. *Dev. Neurobiol.* 72:537. 10.1002/dneu.20880 21312343 PMC3157589

[B32] Diaz C. M. Macnab L. T. Williams S. M. Sullivan R. K. P. Pow D. V. (2007). EAAT1 and d-serine expression are early features of human retinal development. *Exp. Eye Res.* 84 876–885. 10.1016/j.exer.2007.01.008 17379211

[B33] Diniz L. P. Almeida J. C. Tortelli V. Lopes C. V. Setti-Perdigão P. Stipursky J. (2012). Astrocyte-induced Synaptogenesis Is Mediated by Transforming Growth Factor β Signaling through Modulation of d-Serine Levels in Cerebral Cortex Neurons *. *J. Biol. Chem.* 287 41432–41445. 10.1074/jbc.M112.380824 23055518 PMC3510841

[B34] Dixon M. A. Greferath U. Fletcher E. L. Jobling A. I. (2021). The Contribution of Microglia to the Development and Maturation of the Visual System. *Front. Cell. Neurosci.* 15:659843. 10.3389/fncel.2021.659843 33967697 PMC8102829

[B35] Dun Y. Duplantier J. Roon P. Martin P. M. Ganapathy V. Smith S. B. (2008). Serine racemase expression and d-serine content are developmentally regulated in neuronal ganglion cells of the retina. *J. Neurochem.* 104 970–978. 10.1111/j.1471-4159.2007.05015.x 17976164

[B36] Egensperger R. Maslim J. Bisti S. Holländer H. Stone J. (1996). Fate of DNA from retinal cells dying during development: uptake by microglia and macroglia (Müller cells). *Dev. Brain Res.* 97 1–8. 10.1016/s0165-3806(96)00119-8 8946048

[B37] Elliott J. Jolicoeur C. Ramamurthy V. Cayouette M. (2008). Ikaros Confers Early Temporal Competence to Mouse Retinal Progenitor Cells. *Neuron* 60 26–39. 10.1016/j.neuron.2008.08.008 18940586

[B38] Eroglu Ç Allen N. J. Susman M. W. O’Rourke N. A. Park C. Y. Özkan E. (2009). Gabapentin Receptor α2δ-1 Is a Neuronal Thrombospondin Receptor Responsible for Excitatory CNS Synaptogenesis. *Cell* 139 380–392. 10.1016/j.cell.2009.09.025 19818485 PMC2791798

[B39] Farhy-Tselnicker I. Boisvert M. M. Liu H. Dowling C. Erikson G. A. Blanco-Suarez E. (2021). Activity-dependent modulation of synapse-regulating genes in astrocytes. *Elife* 10:70514. 10.7554/eLife.70514 34494546 PMC8497060

[B40] Ford K. J. Félix A. L. Feller M. B. (2012). Cellular Mechanisms Underlying Spatiotemporal Features of Cholinergic Retinal Waves. *J. Neurosci.* 32 850–863. 10.1523/JNEUROSCI.5309-12.2012 22262883 PMC3311224

[B41] Francisco-Morcillo J. Hidalgo-Sánchez M. Martín-Partido G. (2004). Spatial and temporal patterns of apoptosis during differentiation of the retina in the turtle. *Anat. Embryol.* 208 289–299. 10.1007/s00429-004-0398-x 15168116

[B42] Franze K. Grosche J. Skatchkov S. Schinkinger S. Foja C. Schild D. (2007). Müller cells are living optical fibers in the vertebrate retina. *Proc. Natl. Acad. Sci. U. S. A*. 104, 8287–8292. 10.1073/pnas.0611180104 17485670 PMC1895942

[B43] Furukawa T. Mukherjee S. Bao Z. Z. Morrow E. M. Cepko C. L. (2000). rax, Hes1, and notch1 Promote the Formation of Müller Glia by Postnatal Retinal Progenitor Cells. *Neuron* 26 383–394. 10.1016/s0896-6273(00)81171-x 10839357

[B44] Furuya T. Pan Z. Kashiwagi K. (2012). Role of retinal glial cell glutamate transporters in retinal ganglion cell survival following stimulation of NMDA receptor. *Curr. Eye Res.* 37 170–178. 10.3109/02713683.2011.645105 22335803

[B45] García M. Forster V. Hicks D. Vecino E. (2002). Effects of Müller glia on cell survival and neuritogenesis in adult porcine retina in vitro. *Investig. Ophthalmol. Vis. Sci.* 43 3735–3743. 12454045

[B46] Georgi S. A. Reh T. A. (2010). Dicer Is Required for the Transition from Early to Late Progenitor State in the Developing Mouse Retina. *J. Neurosci.* 30:4048. 10.1523/JNEUROSCI.4982-09.2010 20237275 PMC2853880

[B47] Goldman D. (2014). Müller glia cell reprogramming and retina regeneration. *Nat. Rev. Neurosci.* 15:431. 10.1038/nrn3723 PMC4249724 24894585

[B48] Goureau O. Rhee K. Yang X.-J. (2004). Ciliary Neurotrophic Factor Promotes Müller Glia Differentiation from the Postnatal Retinal Progenitor Pool. *Dev. Neurosci.* 26:359. 10.1159/000082278 15855765 PMC7050730

[B49] Guidry C. Bradley K. M. King J. L. (2003). Tractional force generation by human müller cells: growth factor responsiveness and integrin receptor involvement. *Invest. Ophthalmol. Vis. Sci.* 44 1355–1363. 10.1167/iovs.02-0046 12601069

[B50] Hansen K. H. Bracken A. P. Pasini D. Dietrich N. Gehani S. S. Monrad A. (2008). A model for transmission of the H3K27me3 epigenetic mark. *Nat. Cell Biol.* 10 1291–1300. 10.1038/ncb1787 18931660

[B51] Harada T. Harada C. Kohsaka S. Wada E. Yoshida K. Ohno S. (2002). Microglia–Müller Glia Cell Interactions Control Neurotrophic Factor Production during Light-Induced Retinal Degeneration. *J. Neurosci.* 22 9228–9236. 12417648 10.1523/JNEUROSCI.22-21-09228.2002 PMC6758038

[B52] Hasel P. Dando O. Jiwaji Z. Baxter P. Todd A. C. Heron S. (2017). Neurons and neuronal activity control gene expression in astrocytes to regulate their development and metabolism. *Nat. Commun.* 8:15132. 10.1038/ncomms15132 PMC5418577 28462931

[B53] Hauck S. M. Suppmann S. Ueffing M. (2003). Proteomic profiling of primary retinal Müller glia cells reveals a shift in expression patterns upon adaptation to in vitro conditions. *Glia* 44 251–263. 10.1002/glia.10292 14603466

[B54] Henrique D. Hirsinger E. Adam J. Roux I. Le Pourquié O. Ish-Horowicz D. (1997). Maintenance of neuroepithelial progenitor cells by Delta–Notch signalling in the embryonic chick retina. *Curr. Biol.* 7 661–670. 10.1016/s0960-9822(06)00293-4 9285721

[B55] Hirrlinger P. G. Pannicke T. Winkler U. Claudepierre T. Varshney S. Schulze C. (2011). Genetic Deletion of Laminin Isoforms β2 and γ3 Induces a Reduction in Kir4.1 and Aquaporin-4 Expression and Function in the Retina. *PLoS One* 6:e16106. 10.1371/journal.pone.0016106 21283711 PMC3025027

[B56] Hojo M. Ohtsuka T. Hashimoto N. Gradwohl G. Guillemot F. Kageyama R. (2000). Glial cell fate specification modulated by the bHLH gene Hes5 in mouse retina. *Development* 127 2515–2522. 10821751 10.1242/dev.127.12.2515

[B57] Hoon M. Okawa H. Santina L. Wong R. O. (2014). Functional Architecture of the Retina: Development and Disease. *Prog. Retin. Eye Res.* 42:44. 10.1016/j.preteyeres.2014.06.003 24984227 PMC4134977

[B58] Hu Q. Zhang L. Wen J. Wang S. Li M. Feng R. (2010). The Egf Receptor-Sox2-Egf Receptor Feedback Loop Positively Regulates the Self-Renewal of Neural Precursor Cells. *Stem Cells* 28 279–286. 10.1002/stem.246 19882665

[B59] Hughes W. F. Lavelle A. (1975). The effects of early tectal lesions on development in the retinal ganglion cell layer of chick embryos. *J. Comp. Neurol.* 163 265–283. 10.1002/cne.901630303 1176640

[B60] Ibáñez I. Bartolomé-Martín D. Piniella D. Giménez C. Zafra F. (2019). Activity dependent internalization of the glutamate transporter GLT-1 requires calcium entry through the NCX sodium/calcium exchanger. *Neurochem. Int.* 123 125–132. 10.1016/j.neuint.2018.03.012 29574129

[B61] Iida A. Iwagawa T. Baba Y. Satoh S. Mochizuki Y. Nakauchi H. (2015). Roles of histone H3K27 trimethylase Ezh2 in retinal proliferation and differentiation. *Dev. Neurobiol.* 75 947–960. 10.1002/dneu.22261 25556712

[B62] Jadhav A. P. Cho S.-H. Cepko C. L. (2006). Notch activity permits retinal cells to progress through multiple progenitor states and acquire a stem cell property. *Proc. Natl. Acad. Sci. U S A.* 103:18998. 10.1073/pnas.0608155103 17148603 PMC1682012

[B63] Keeley P. Eglen S. Reese B. (2020). From random to regular: variation in the patterning of retinal mosaics. *J. Comp. Neurol*. 528, 2135–2160. 10.1002/cne.24880 32026463 PMC7368823

[B64] Kerschensteiner D. (2016). Glutamatergic retinal waves. *Front. Neural Circuits* 10:38. 10.3389/fncir.2016.00038 27242446 PMC4861735

[B65] Koh S. Chen W. J. Dejneka N. S. Harris I. R. Lu B. Girman S. (2018). Subretinal Human Umbilical Tissue-Derived Cell Transplantation Preserves Retinal Synaptic Connectivity and Attenuates Müller Glial Reactivity. *J. Neurosci.* 38 2923–2943. 10.1523/JNEUROSCI.1532-17.2018 29431645 PMC5864147

[B66] Koh S. Roy S. Eroglu O. Strader S. Chen W. J. Kay J. N. (2019). Thrombospondin-1 Promotes Circuit-Specific Synapse Formation via β1-Integrin. *bioRxiv* 2019:866590. 10.1101/866590

[B67] Kohn A. Rutkowski T. P. Liu Z. Mirando A. J. Zuscik M. J. O’Keefe R. J. (2015). Notch signaling controls chondrocyte hypertrophy via indirect regulation of Sox9. *Bone Res.* 3 1–12. 10.1038/boneres.2015.21 26558140 PMC4640428

[B68] Kucukdereli H. Allen N. J. Lee A. T. Feng A. Ozlu M. I. Conatser L. M. (2011). Control of excitatory CNS synaptogenesis by astrocyte-secreted proteins hevin and SPARC. *Proc. Natl. Acad. Sci. U S A.* 108 E440–E449. 10.1073/pnas.1104977108 21788491 PMC3156217

[B69] La Torre A. Georgi S. Reh T. A. (2013). Conserved microRNA pathway regulates developmental timing of retinal neurogenesis. *Proc. Natl. Acad. Sci. U S A.* 110 E2362. 10.1073/pnas.1301837110 23754433 PMC3696811

[B70] La Vail M. M. Rapaport D. H. Rakic P. (1991). Cytogenesis in the monkey retina. *J. Comp. Neurol.* 309 86–114. 10.1002/cne.903090107 1894769

[B71] Lamas M. Lee-Rivera I. Ramírez M. López-Colomé A. M. (2007). d-Serine regulates CREB phosphorylation induced by NMDA receptor activation in Müller glia from the retina. *Neurosci. Lett.* 427 55–60. 10.1016/j.neulet.2007.09.009 17920195

[B72] Lange J. Yafai Y. Noack A. Yang X. M. Munk A.-B. Krohn S. (2012). The axon guidance molecule Netrin-4 is expressed by Müller cells and contributes to angiogenesis in the retina. *Glia* 60 1567–1578. 10.1002/glia.22376 22777897

[B73] Lasorella A. Uo T. Iavarone A. (2002). Id proteins at the cross-road of development and cancer. *Oncogene* 20 8326–8333. 10.1038/sj.onc.1205093 11840325

[B74] Lavialle M. Aumann G. Anlauf E. Pröls F. Arpin M. Derouiche A. (2011). Structural plasticity of perisynaptic astrocyte processes involves ezrin and metabotropic glutamate receptors. *Proc. Natl. Acad. Sci.* 108 12915–12919. 10.1073/pnas.1100957108 21753079 PMC3150955

[B75] Lee C. W. Eglen S. J. Wong R. O. L. (2002). Segregation of ON and OFF retinogeniculate connectivity directed by patterned spontaneous activity. *J. Neurophysiol.* 88 2311–2321. 10.1152/jn.00372.2002 12424272

[B76] Levine E. M. Close J. Fero M. Ostrovsky A. Reh T. A. (2000). p27Kip1 Regulates Cell Cycle Withdrawal of Late Multipotent Progenitor Cells in the Mammalian Retina. *Dev. Biol.* 219 299–314. 10.1006/dbio.2000.9622 10694424

[B77] Li F. Jiang D. Samuel M. A. (2019). Microglia in the developing retina. *Neural Dev.* 14 1–13. 31888774 10.1186/s13064-019-0137-x PMC6938006

[B78] Lillien L. (1995). Changes in retinal cell fate induced by overexpression of EGF receptor. *Nature* 377 158–161. 10.1038/377158a0 7675083

[B79] Lillien L. Wancio D. (1998). Changes in Epidermal Growth Factor Receptor Expression and Competence to Generate Glia Regulate Timing and Choice of Differentiation in the Retina. *Mol. Cell. Neurosci.* 10 296–308. 10.1006/mcne.1997.0659 9618220

[B80] Livesey F. J. Cepko C. L. (2001). Vertebrate neural cell-fate determination: Lessons from the retina. *Nat. Rev. Neurosci.* 2 109–118. 10.1038/35053522 11252990

[B81] López-Colomé A. M. López E. Mendez-Flores O. G. Ortega A. (2016). Glutamate Receptor Stimulation Up-Regulates Glutamate Uptake in Human Müller Glia Cells. *Neurochem. Res.* 41 1797–1805. 10.1007/s11064-016-1895-z 27017513

[B82] Louvi A. Artavanis-Tsakonas S. (2006). Notch signalling in vertebrate neural development. *Nat. Rev. Neurosci.* 7 93–102. 10.1038/nrn1847 16429119

[B83] MacDonald R. B. Randlett O. Oswald J. Yoshimatsu T. Franze K. Harris W. A. (2015). Müller glia provide essential tensile strength to the developing retina. *J. Cell Biol.* 210 1075–1083. 10.1083/jcb.201503115 26416961 PMC4586739

[B84] Marín-Teva J. L. Cuadros M. A. Calvente R. Almendros A. Navascués J. (1999). Naturally Occurring Cell Death and Migration of Microglial Precursors in the Quail Retina During Normal Development. *J. Comp. Neurol.* 412 255–275. 10.1002/(sici)1096-9861(19990920)412:2<255::aid-cne6>3.0.co;2-h 10441755

[B85] Melo J. de Clark B. S. Blackshaw S. (2016a). Multiple intrinsic factors act in concert with Lhx2 to direct retinal gliogenesis. *Sci. Rep.* 6:32757. 10.1038/srep32757 27605455 PMC5015061

[B86] Melo J. de Zibetti C. Clark B. S. Hwang W. Miranda-Angulo A. L. Qian J. (2016b). Lhx2 Is an Essential Factor for Retinal Gliogenesis and Notch Signaling. *J. Neurosci.* 36:2391. 10.1523/JNEUROSCI.3145-15.2016 26911688 PMC4764661

[B87] Metea M. R. Newman E. A. (2006). Glial Cells Dilate and Constrict Blood Vessels: A Mechanism of Neurovascular Coupling. *J. Neurosci.* 26 2862–2870. 10.1523/JNEUROSCI.4048-05.2006 16540563 PMC2270788

[B88] Miller S. J. Philips T. Kim N. Dastgheyb R. Chen Z. Hsieh Y. C. (2019). Molecularly defined cortical astroglia subpopulation modulates neurons via secretion of Norrin. *Nat. Neurosci.* 22 741–752. 10.1038/s41593-019-0366-7 30936556 PMC6551209

[B89] Mizeracka K. DeMaso C. R. Cepko C. L. (2013). Notch1 is required in newly postmitotic cells to inhibit the rod photoreceptor fate. *Development* 140 3188–3197. 10.1242/dev.090696 23824579 PMC3931735

[B90] Molotkov D. Zobova S. Arcas J. M. Khiroug L. (2013). Calcium-induced outgrowth of astrocytic peripheral processes requires actin binding by Profilin-1. *Cell Calcium* 53 338–348. 10.1016/j.ceca.2013.03.001 23578580

[B91] Müller H. (1851). Zur Histologie der Netzhaut. *Zeitschrift für Wiss Zool* 3 234–237.

[B92] Musada G. R. Dvoriantchikova G. Myer C. Ivanov D. Bhattacharya S. K. Hackam A. S. (2020). The effect of extrinsic Wnt/β-catenin signaling in Muller glia on retinal ganglion cell neurite growth. *Dev. Neurobiol.* 80 98–110. 10.1002/dneu.22741 32267608 PMC7377969

[B93] Nagai J. Rajbhandari A. K. Gangwani M. R. Hachisuka A. Coppola G. Masmanidis S. C. (2019). Hyperactivity with disrupted attention by activation of an astrocyte synaptogenic cue. *Cell* 177:1280. 10.1016/j.cell.2019.03.019 31031006 PMC6526045

[B94] Nagashima M. Hadidjojo J. Barthel L. K. Lubensky D. K. Raymond P. A. (2017). Anisotropic Müller glial scaffolding supports a multiplex lattice mosaic of photoreceptors in zebrafish retina. *Neural Dev.* 12 1–20. 10.1186/s13064-017-0096-z 29141686 PMC5688757

[B95] Nelson B. R. Ueki Y. Reardon S. Karl M. O. Georgi S. Hartman B. H. (2011). Genome-Wide Analysis of Müller Glial Differentiation Reveals a Requirement for Notch Signaling in Postmitotic Cells to Maintain the Glial Fate. *PLoS One* 6:e22817. 10.1371/journal.pone.0022817 21829655 PMC3149061

[B96] Nomura-Komoike K. Saitoh F. Fujieda H. (2020). Phosphatidylserine recognition and Rac1 activation are required for Müller glia proliferation, gliosis and phagocytosis after retinal injury. *Sci. Rep.* 10 1–11. 10.1038/s41598-020-58424-6 32001733 PMC6992786

[B97] Paisley C. E. Kay J. N. (2021). Seeing stars: Development and function of retinal astrocytes. *Dev. Biol.* 478 144–154. 10.1016/j.ydbio.2021.07.007 34260962 PMC8542354

[B98] Pannicke T. Bringmann A. Reichenbach A. (2002). Electrophysiological characterization of retinal Müller glial cells from mouse during postnatal development: Comparison with rabbit cells. *Glia* 38 268–272. 10.1002/glia.10068 11968064

[B99] Patel A. K. Surapaneni K. Yi H. Nakamura R. E. I. Karli S. Z. Syeda S. (2015). Activation of Wnt/β-catenin signaling in Muller glia protects photoreceptors in a mouse model of inherited retinal degeneration. *Neuropharmacology* 0:1. 10.1016/j.neuropharm.2014.11.015 25486619 PMC4312718

[B100] Pena J. Dulger N. Singh T. Zhou J. Majeska R. Redenti S. (2018). Controlled microenvironments to evaluate chemotactic properties of cultured Müller glia. *Exp. Eye Res.* 173 129–137. 10.1016/j.exer.2018.05.005 29753729 PMC6054825

[B101] Peng Y. R. Shekhar K. Yan W. Herrmann D. Sappington A. Bryman G. S. (2019). Molecular Classification and Comparative Taxonomics of Foveal and Peripheral Cells in Primate Retina. *Cell* 176 1222.e–1237.e. 10.1016/j.cell.2019.01.004 30712875 PMC6424338

[B102] Phillips J. B. Blanco-Sanchez B. Lentz J. J. Tallafuss A. Khanobdee K. Sampath S. (2011). Harmonin (Ush1c) is required in zebrafish Müller glial cells for photoreceptor synaptic development and function. *Dis. Model. Mech.* 4:786. 10.1242/dmm.006429 21757509 PMC3209648

[B103] Poché R. A. Furuta Y. Chaboissier M.-C. Schedl A. Behringer R. R. (2008). Sox9 Is Expressed in Mouse Multipotent Retinal Progenitor Cells and Functions in Müller Glial Cell Development. *J. Comp. Neurol.* 510:237. 10.1002/cne.21746 18626943 PMC4412477

[B104] Pow D. V. Barnett N. L. (1999). Changing patterns of spatial buffering of glutamate in developing rat retinae are mediated by the Müller cell glutamate transporter GLAST. *Cell Tissue Res.* 297 57–66. 10.1007/s004410051333 10398883

[B105] Prada C. Puga J. Perez,-Mendez Lopez R. Ramirez G. (1991). Spatial and Temporal Patterns of Neurogenesis in the Chick Retina. *Eur. J. Neurosci.* 3 559–569. 10.1111/j.1460-9568.1991.tb00843.x 12106488

[B106] Qian Z. Bilderback T. R. Barmack N. H. (2008). Acyl coenzyme A-binding protein (ACBP) is phosphorylated and secreted by retinal Müller astrocytes following protein kinase C activation. *J. Neurochem.* 105 1287–1299. 10.1111/j.1471-4159.2008.05229.x 18194441

[B107] Quintero H. Lamas M. (2018). microRNA expression in the neural retina: Focus on Müller glia. *J. Neurosci. Res.* 96 362–370. 29030949 10.1002/jnr.24181

[B108] Ramon y Cajal S. (1893). La rétine des vertébrés. *Cellule* 9 120–258.

[B109] Randlett O. MacDonald R. B. Yoshimatsu T. Almeida A. D. Suzuki S. C. Wong R. O. (2013). Cellular Requirements for Building a Retinal Neuropil. *Cell Rep.* 3:282. 10.1016/j.celrep.2013.01.020 23416047 PMC3607253

[B110] Reese B. E. Keeley P. W. (2015). Design principles and developmental mechanisms underlying retinal mosaics. *Biol. Rev. Camb. Philos. Soc.* 90:854. 10.1111/brv.12139 25109780 PMC4320990

[B111] Reichenbach A. Bringmann A. (2013). New functions of Müller cells. *Glia* 61 651–678. 10.1002/glia.22477 23440929

[B112] Reichenbach A. Bringmann A. (2016). Role of Purines in Müller Glia. *J. Ocul. Pharmacol. Ther.* 32 518–533. 10.1089/jop.2016.0131 27754822

[B113] Reichenbach A. Reichelt W. (1986). Postnatal development of radial glial (Müller) cells of the rabbit retina. *Neurosci. Lett.* 71 125–130. 10.1016/0304-3940(86)90545-8 3785738

[B114] Ribot J. Breton R. Calvo C.-F. Moulard J. Ezan P. Zapata J. (2021). Astrocytes close the mouse critical period for visual plasticity. *Science* 373 77–81. 10.1126/science.abf5273 34210880

[B115] Rich K. A. Figueroa S. L. Zhan Y. Blanks J. C. (1995). Effects of Müller cell disruption on mouse photoreceptor cell development. *Exp. Eye Res.* 61 235–248. 10.1016/s0014-4835(05)80043-0 7556487

[B116] Richier B. Vijandi C. Mackensen S. Salecker I. (2017). Lapsyn controls branch extension and positioning of astrocyte-like glia in the Drosophila optic lobe. *Nat. Commun.* 8 1–17. 10.1038/s41467-017-00384-z 28827667 PMC5567088

[B117] Rillich K. Gentsch J. Reichenbach A. Bringmann A. Weick M. (2009). Light stimulation evokes two different calcium responses in Müller glial cells of the guinea pig retina. *Eur. J. Neurosci.* 29 1165–1176. 10.1111/j.1460-9568.2009.06682.x 19302152

[B118] Rosa J. M. Bos R. Sack G. S. Fortuny C. Agarwal A. Bergles D. E. (2015). Neuron-glia signaling in developing retina mediated by neurotransmitter spillover. *Elife* 4 1–20. 10.7554/eLife.09590 26274565 PMC4566075

[B119] Ruzafa N. Pereiro X. Lepper M. F. Hauck S. M. Vecino E. (2018). A Proteomics Approach to Identify Candidate Proteins Secreted by Müller Glia that Protect Ganglion Cells in the Retina. *Proteomics* 18:1700321. 10.1002/pmic.201700321 29645351

[B120] Saint-Geniez M. Maharaj A. S. R. Walshe T. E. Tucker B. A. Sekiyama E. Kurihara T. (2008). Endogenous VEGF Is Required for Visual Function: Evidence for a Survival Role on Müller Cells and Photoreceptors. *PLoS One* 3:e3554. 10.1371/journal.pone.0003554 18978936 PMC2571983

[B121] Sardar Pasha S. P. B. Münch R. Schäfer P. Oertel P. Sykes A. M. Zhu Y. (2017). Retinal cell death dependent reactive proliferative gliosis in the mouse retina. *Sci. Rep.* 7 1–16. 10.1038/s41598-017-09743-8 28842607 PMC5572737

[B122] Satow T. Bae S.-K. Inoue T. Inoue C. Miyoshi G. Tomita K. (2001). The Basic Helix-Loop-Helix Gene hesr2 Promotes Gliogenesis in Mouse Retina. *J. Neurosci.* 21 1265–1273. 10.1523/JNEUROSCI.21-04-01265.2001 11160397 PMC6762251

[B123] Scheer N. Groth A. Hans S. Campos-Ortega J. A. (2001). An instructive function for Notch in promoting gliogenesis in the zebrafish retina. *Development* 128 1099–1107. 10.1242/dev.128.7.1099 11245575

[B124] Schell M. J. Brady R. O. Molliver M. E. Snyder S. H. (1997). D-Serine as a Neuromodulator: Regional and Developmental Localizations in Rat Brain Glia Resemble NMDA Receptors. *J Neurosci.* 17 1604–1615. 10.1523/JNEUROSCI.17-05-01604.1997 9030620 PMC6573391

[B125] Scott R. S. McMahon E. J. Pop S. M. Reap E. A. Caricchio R. Cohen P. L. (2001). Phagocytosis and clearance of apoptotic cells is mediated by MER. *Nat.* 411 207–211. 10.1038/35075603 11346799

[B126] Seitz R. Hackl S. Seibuchner T. Tamm E. R. Ohlmann A. (2010). Norrin Mediates Neuroprotective Effects on Retinal Ganglion Cells via Activation of the Wnt/β-Catenin Signaling Pathway and the Induction of Neuroprotective Growth Factors in Müller Cells. *J. Neurosci.* 30 5998–6010. 10.1523/JNEUROSCI.0730-10.2010 20427659 PMC6632606

[B127] Shekhar K. Sanes J. R. (2021). Generating and Using Transcriptomically Based Retinal Cell Atlases. *Annu. Rev. Vis. Sci.* 7 43–72. 10.1146/annurev-vision-032621 34228938

[B128] Shekhar K. Lapan S. W. Whitney I. E. Tran N. M. Macosko E. Z. Kowalczyk M. (2016). Comprehensive Classification of Retinal Bipolar Neurons by Single-Cell Transcriptomics. *Cell* 166 1308.e–1323.e. 10.1016/j.cell.2016.07.054 27565351 PMC5003425

[B129] Stone J. Itin A. Alon T. Pe’er J. Gnessin H. Chan-Ling T. (1995). Development of Retinal Vasculature Is Mediated by Hypoxia-Induced Vascular Endothelial Growth Factor (VEGF) Expression by Neuroglia. *J. Neurosci.* 15 4738–4747. 10.1523/JNEUROSCI.15-07-04738.1995 7623107 PMC6577882

[B130] Stork T. Sheehan A. Tasdemir-Yilmaz O. E. Freeman M. R. (2014). Neuron-Glia Interactions through the Heartless FGF Receptor Signaling Pathway Mediate Morphogenesis of Drosophila Astrocytes. *Neuron* 83 388–403. 10.1016/j.neuron.2014.06.026 25033182 PMC4124900

[B131] Taguchi M. Shinozaki Y. Kashiwagi K. Shigetomi E. Robaye B. Koizumi S. (2016). Müller cell-mediated neurite outgrowth of the retinal ganglion cells via P2Y6 receptor signals. *J. Neurochem.* 136 741–751. 10.1111/jnc.13427 26560804

[B132] Tan C. X. Burrus Lane C. J. Eroglu C. (2021). Role of astrocytes in synapse formation and maturation. *Curr. Top. Dev. Biol.* 142 371–407. 10.1016/bs.ctdb.2020.12.010 33706922

[B133] Taylor S. Srinivasan B. Wordinger R. J. Roque R. S. (2003). Glutamate stimulates neurotrophin expression in cultured Müller cells. *Mol. Brain Res.* 111 189–197. 10.1016/s0169-328x(03)00030-5 12654519

[B134] Todd L. Squires N. Suarez L. Fischer A. J. (2016). Jak/Stat signaling regulates the proliferation and neurogenic potential of Müller glia-derived progenitor cells in the avian retina. *Sci. Rep.* 6 1–16. 10.1038/srep35703 27759082 PMC5069623

[B135] Toerne C. von Menzler J. Ly A. Senninger N. Ueffing M. Hauck S. M. (2014). Identification of a Novel Neurotrophic Factor from Primary Retinal Müller Cells Using Stable Isotope Labeling by Amino Acids in Cell Culture (SILAC). *Mol. Cell. Proteomics* 13:2371. 10.1074/mcp.M113.033613 24925906 PMC4159655

[B136] Too L. K. Simunovic M. P. (2021). Retinal Stem/Progenitor Cells Derived From Adult Müller Glia for the Treatment of Retinal Degeneration. *Front. Cell Dev. Biol.* 0:2683. 10.3389/fcell.2021.749131 34660607 PMC8511496

[B137] Raju T. R. Bennett M. R. (1986). Retinal ganglion cell survival requirements: a major but transient dependence on Müller glia during development. *Brain Res.* 383 165–176. 10.1016/0006-8993(86)90017-x 3768687

[B138] Tran M. D. Neary J. T. (2006). Purinergic signaling induces thrombospondin-1 expression in astrocytes. *Proc. Natl. Acad. Sci.* 103 9321–9326. 10.1073/pnas.0603146103 16754856 PMC1482608

[B139] Tremblay M. Fugère V. Tsui J. Schohl A. Tavakoli A. Travençolo B. A. N. (2009). Regulation of Radial Glial Motility by Visual Experience. *J. Neurosci.* 29:14066. 10.1523/JNEUROSCI.3542-09.2009 19906955 PMC6665059

[B140] Tworig J. M. Coate C. Feller M. B. (2021). Excitatory neurotransmission activates compartmentalized calcium transients in Müller glia without affecting lateral process motility. *Elife* 2021:73202. 10.7554/eLife.73202 34913435 PMC8806189

[B141] Uckermann O. Vargová L. Ulbricht E. Klaus C. Weick M. Rillich K. (2004). Glutamate-evoked alterations of glial and neuronal cell morphology in the guinea pig retina. *J. Neurosci.* 24 10149–10158. 10.1523/JNEUROSCI.3203-04.2004 15537885 PMC6730174

[B142] Ueki Y. Reh T. A. (2013). EGF Stimulates Müller Glial Proliferation via a BMP Dependent Mechanism. *Glia* 61:778. 10.1002/glia.22472 23362023 PMC4007577

[B143] Ueki Y. Wilken M. S. Cox K. E. Chipman L. B. Bermingham-McDonogh O. Reh T. A. (2015). A transient wave of BMP signaling in the retina is necessary for Müller glial differentiation. *Development* 142:533. 10.1242/dev.118745 25605781 PMC4302996

[B144] Ueno K. Iwagawa T. Ochiai G. Koso H. Nakauchi H. Nagasaki M. (2017). Analysis of Müller glia specific genes and their histone modification using Hes1-promoter driven EGFP expressing mouse. *Sci. Rep.* 7:3578. 10.1038/s41598-017-03874-8 PMC5472600 28620206

[B145] Unterlauft J. D. Claudepierre T. Schmidt M. Müller K. Yafai Y. Wiedemann P. (2014). Enhanced survival of retinal ganglion cells is mediated by Müller glial cell-derived PEDF. *Exp. Eye Res.* 127 206–214. 10.1016/j.exer.2014.08.004 25128578

[B146] Uribe R. A. Gross J. M. (2010). Id2a influences neuron and glia formation in the zebrafish retina by modulating retinoblast cell cycle kinetics. *Development* 137 3763–3774. 10.1242/dev.050484 20943708 PMC3049276

[B147] Vincent A. J. Lau P. W. Roskams A. J. (2008). SPARC is expressed by macroglia and microglia in the developing and mature nervous system. *Dev. Dyn.* 237 1449–1462. 10.1002/dvdy.21495 18366138

[B148] Wang J. O’Sullivan M. L. Mukherjee D. Puñal V. M. Farsiu S. Kay J. N. (2017). Anatomy and spatial organization of Müller glia in mouse retina. *J. Comp. Neurol.* 525 1759–1777. 10.1002/cne.24153 27997986 PMC5542564

[B149] Wang M. Ma W. Zhao L. Fariss R. N. Wong W. T. (2011). Adaptive Müller cell responses to microglial activation mediate neuroprotection and coordinate inflammation in the retina. *J. Neuroinflamm.* 8 1–19. 10.1186/1742-2094-8-173 22152278 PMC3251543

[B150] Wang Y. P. Dakubo G. Howley P. Campsall K. D. Mazarolle C. J. Shiga S. A. (2002). Development of normal retinal organization depends on Sonic hedgehog signaling from ganglion cells. *Nat. Neurosci.* 5 831–832. 10.1038/nn911 12195432

[B151] Williams P. R. Suzuki S. C. Yoshimatsu T. Lawrence O. T. Waldron S. J. Parsons M. J. (2010). In vivo development of outer retinal synapses in the absence of glial contact. *J. Neurosci.* 30 11951–11961. 10.1523/JNEUROSCI.3391-10.2010 20826659 PMC2946228

[B152] Williams S. M. Diaz C. M. Macnab L. T. Sullivan R. K. P. Pow D. V. (2006). Immunocytochemical analysis of D-serine distribution in the mammalian brain reveals novel anatomical compartmentalizations in glia and neurons. *Glia* 53 401–411. 10.1002/glia.20300 16342169

[B153] Wohl S. G. Jorstad N. L. Levine E. M. Reh T. A. (2017). Müller glial microRNAs are required for the maintenance of glial homeostasis and retinal architecture. *Nat. Commun.* 8 1–15. 10.1038/s41467-017-01624-y 29150673 PMC5693933

[B154] Wohl S. G. Reh T. A. Christenson L. K. Jimenez D. F. Digicaylioglu M. (2016). The microRNA expression profile of mouse Müller glia in vivo and in vitro. *Sci. Rep.* 6:35423. 10.1038/srep35423 PMC5064377 27739496

[B155] Wong L. L. Rapaport D. H. (2009). Defining retinal progenitor cell competence in Xenopus laevis by clonal analysis. *Development* 136 1707–1715. 10.1242/dev.027607 19395642 PMC2673759

[B156] Wurm A. Pannicke T. Iandiev I. Wiedemann P. Reichenbach A. Bringmann A. (2006). The developmental expression of K+ channels in retinal glial cells is associated with a decrease of osmotic cell swelling. *Glia* 54 411–423. 10.1002/glia.20391 16886204

[B157] Xie H. Pallero M. A. Gupta K. Chang P. Ware M. F. Witke W. (1998). EGF receptor regulation of cell motility: EGF induces disassembly of focal adhesions independently of the motility-associated PLCγ signaling pathway. *J. Cell Sci.* 111 615–624. 10.1242/jcs.111.5.615 9454735

[B158] Xu H. P. Burbridge T. J. Ye M. Chen M. Ge X. Zhou Z. J. (2016). Retinal Wave Patterns Are Governed by Mutual Excitation among Starburst Amacrine Cells and Drive the Refinement and Maintenance of Visual Circuits. *J. Neurosci.* 36 3871–3886. 10.1523/JNEUROSCI.3549-15.2016 27030771 PMC4812142

[B159] Xu Q. Wang Y. Dabdoub A. Smallwood P. M. Williams J. Woods C. (2004). Vascular Development in the Retina and Inner Ear: Control by Norrin and Frizzled-4, a High-Affinity Ligand-Receptor Pair. *Cell* 116 883–895. 10.1016/s0092-8674(04)00216-8 15035989

[B160] Yamagata M. Yan W. Sanes J. R. (2021). A cell atlas of the chick retina based on single-cell transcriptomics. *Elife* 10 1–39. 10.7554/eLife.63907 33393903 PMC7837701

[B161] Yan W. Peng Y.-R. van Zyl T. Regev A. Shekhar K. Juric D. (2020). Cell Atlas of The Human Fovea and Peripheral Retina. *Sci. Rep.* 10 1–17. 10.1038/s41598-020-66092-9 32555229 PMC7299956

[B162] Ye X. Wang Y. Cahill H. Yu M. Badea T. C. Smallwood P. M. (2009). Norrin, Frizzled-4, and Lrp5 Signaling in Endothelial Cells Controls a Genetic Program for Retinal Vascularization. *Cell* 139 285–298. 10.1016/j.cell.2009.07.047 19837032 PMC2779707

[B163] Yoshida S. Sotozono C. Ikeda T. Kinoshita S. (2001). Interleukin-6 (IL-6) production by cytokine-stimulated human Müller cells. *Curr. Eye Res.* 22 341–347. 10.1076/ceyr.22.5.341.5498 11600934

[B164] Young R. W. (1985). Cell differentiation in the retina of the mouse. *Anat. Rec.* 212 199–205. 3842042 10.1002/ar.1092120215

[B165] Zhang J. Taylor R. J. La Torre A. Wilken M. S. Cox K. E. Reh T. A. (2015). Ezh2 maintains retinal progenitor proliferation, transcriptional integrity, and the timing of late differentiation. *Dev. Biol.* 403 128–138. 10.1016/j.ydbio.2015.05.010 25989023 PMC4469612

[B166] Zhang R. W. Du W. J. Prober D. A. Du J. L. (2019). Müller Glial Cells Participate in Retinal Waves via Glutamate Transporters and AMPA Receptors. *Cell Rep.* 27 2871.e–2880.e. 10.1016/j.celrep.2019.05.011 31167134 PMC6659749

[B167] Zheng-Zheng B. Cepko C. L. (1997). The expression and function of Notch pathway genes in the developing rat eye. *J. Neurosci.* 17 1425–1434. 10.1523/JNEUROSCI.17-04-01425.1997 9006984 PMC6793727

